# Similia vs. Idem

**Published:** 1881-03

**Authors:** C. Lippe

**Affiliations:** New York City


### SIMILIA VERSUS IDEM.

BY C. LIPPE, M.D., NEW YORK CITY.

A very intelligent lady once put the question to me, if it was not homœopathic practice to give opium to a person given to opium eating. My reply was that opium was not necessarily the antidote because opium had been taken; but the proper remedy must be selected according to the conditions of the case to be treated. Among some of the profession there appears to be some obscurity on this point, and the fact of having removed some difficulties by the administration of the drug which produced the mischief, has led to a sort of generalizing on this point.

I will illustrate by giving two cases. The first was a young man who had contracted gonorrhœa and was treated by strychnia injections. He came to me with the most terrible strangury, constant urging to urinate, only passing a few drops with great straining and pain, the gonorrhœal discharge suppressed; he had been in this condition for twelve hours. The whole case called for *Nux*, and he was given *one* dose 71 m Fincke and within half an hour he passed some urine and in a short time that difficulty was removed with the restoration of the discharge. The gonorrhœa was very obstinate and yielded only after a month's treatment, but there was no more strangury.

The second case was one of opium eating. A young woman, under severe mental strain, was unable to sleep, and the attending physician gave her opium in some form and she became addicted to the habit. Her friends finding her mental condition becoming changed under the influence of the drug persuaded her to put herself under my care. Her condition was of entire sleeplessness, starting at the least noise, quiet and taciturn when not under the influence of the drug, headache sharp and shooting with intolerance of light and noise, appetite gone and constipated. Here was a fine opportunity for the exhibition of opium potentized and, as an experiment, it was given. She came back to me in a few days, of course no better, and begged for relief or she must take her pills of opium. The case was a clear one for *Bellad.*, and she received a dose of that remedy 60 m Fincke. A better night followed and she soon regained her normal condition, and now, at the end of a year, she has taken no opium or medicine of any kind. About three days after the dose of *Bellad.* a profuse watery coryza broke out, but on looking over the proving of the remedy, was satisfied that it was caused by the drug, so gave no more medicine. The law we can deduce is that in these cases, as in all others, the curative remedy is the one in which the totality of the symptoms of the drug and the patient correspond. We *must* individualize, not generalize.

